# Supplementary material for: Triacylglycerols and Other Lipids Profiling of Hemp By-Products
Source: Molecules. 2022 Apr 5;27(7):2339. doi: 10.3390/molecules27072339 (PMC9000728; doi:10.3390/molecules27072339)
Supplement: Supplementary file 1 [file molecules-27-02339-s001.zip › molecules-1659218-supplementary.pdf]

SUPPLEMENTARY DOCUMENT

Triacylglycerols and Other Lipids Profiling of Hemp By-Products

Arjun H. Banskota *, Alysson Jones, Joseph P. M. Hui and Roumiana Stefanova

Aquatic and Crop Resource Development Research Centre, National Research Council Canada, 1411 Oxford Street, Halifax, Nova Scotia, B3H 3Z1, Canada
*Correspondence: Arjun.Banskota@nrc-cnrc.gc.ca; Tel.: 1-902-426-7372

Abstract: Hemp seed by-products namely hemp cake (hemp meal) and hemp hulls were studied for their lipid content and composition. Total lipid content of hemp cake and hemp hulls were 13.1% and 17.5%, respectively. Oil extraction yields using hexane, on the other hand, were much lower in hemp cake (7.4%) and hemp hulls (12.1%). Oil derived from both hemp seeds and by-products were primarily composed of neutral lipids (>97.1%) mainly triacylglycerols (TAGs) determined by SPE and confirmed by NMR study. Linoleic acid was the major fatty acid present in oils derived from hemp by-products covering almost 55% followed by α-linolenic acid covering around 18% of the total fatty acids. For the first time 47 intact TAGs were identified in the hemp oils using UPLC-HRMS. Among them TAGs with fatty acid acyl chain 18:3/18:2/18:2 and 18:3/18:2/18:1 were the major ones, followed by TAGs with fatty acid acyl chain of 18:3/18:3/18:2, 18:2/18:2/16:0, 18:2/18:2/18:1, 18:3/18:2.18:0, 18:2/18:2/18:0, 18:2/18:1/18:1 and 18:3/18:2:16:0. Besides TAGs, low levels of terpenes, carotenoids and cannabidiolic acid were also detected in the oils. Moreover, the oils extracted from hemp by-products possessed a dose-dependent DPPH radical scavenging property and their potencies were in a similar range compared to other vegetable oils.

Keywords: hemp seed by-products; hemp seed oil; hemp cake; hemp hulls; triacylglycerols; fatty acids; DPPH radical scavenging activity.

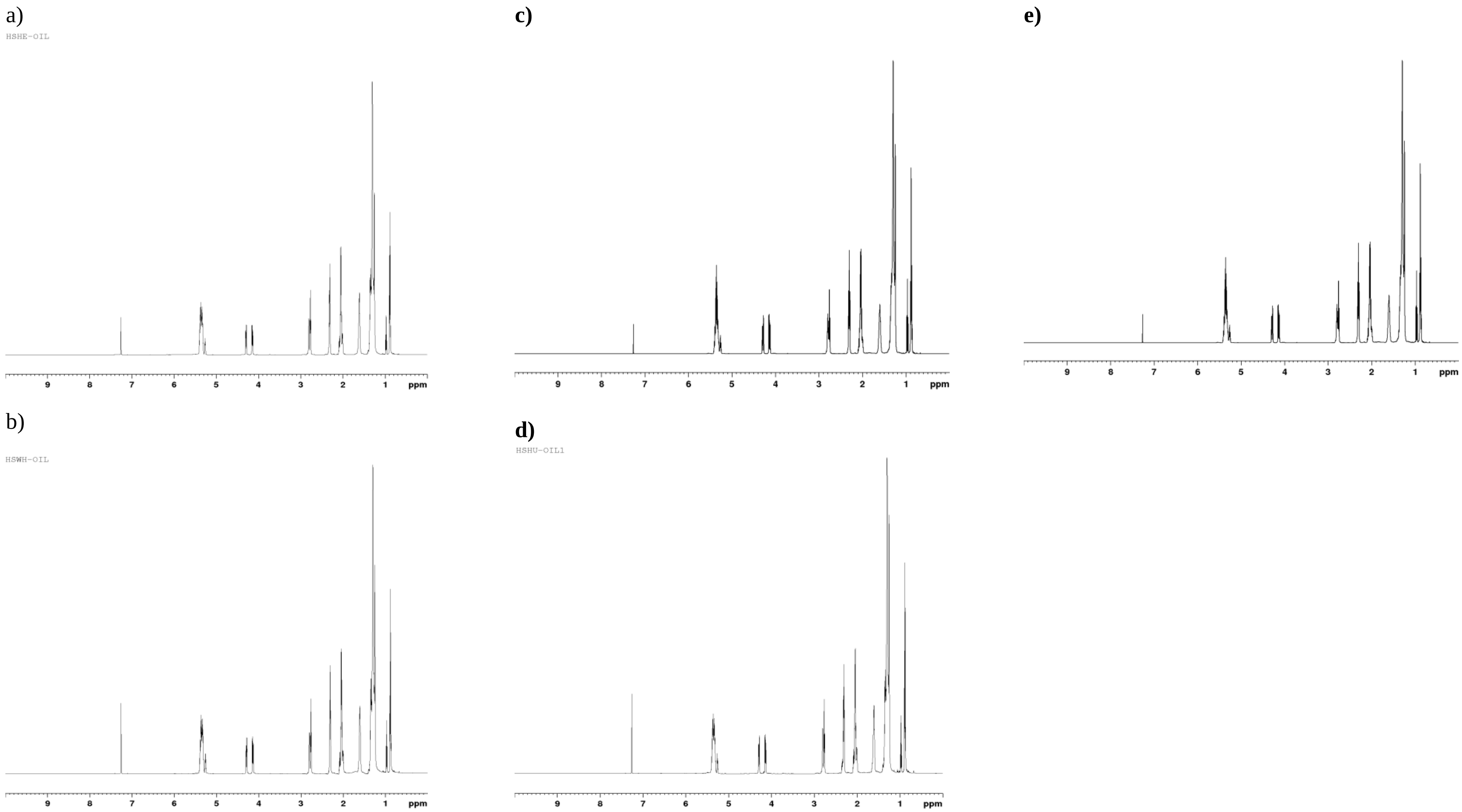

Figure S1. ^{1}H NMR spectrum of oil extracted from and hemp seeds and hemp by-products recorded in 500/700 MHz NMR Spectrometer. a) hemp hearts, b) whole hemp seeds, c) hemp cake, d) hemp seed hulls and e) cold pressed hemp oil.

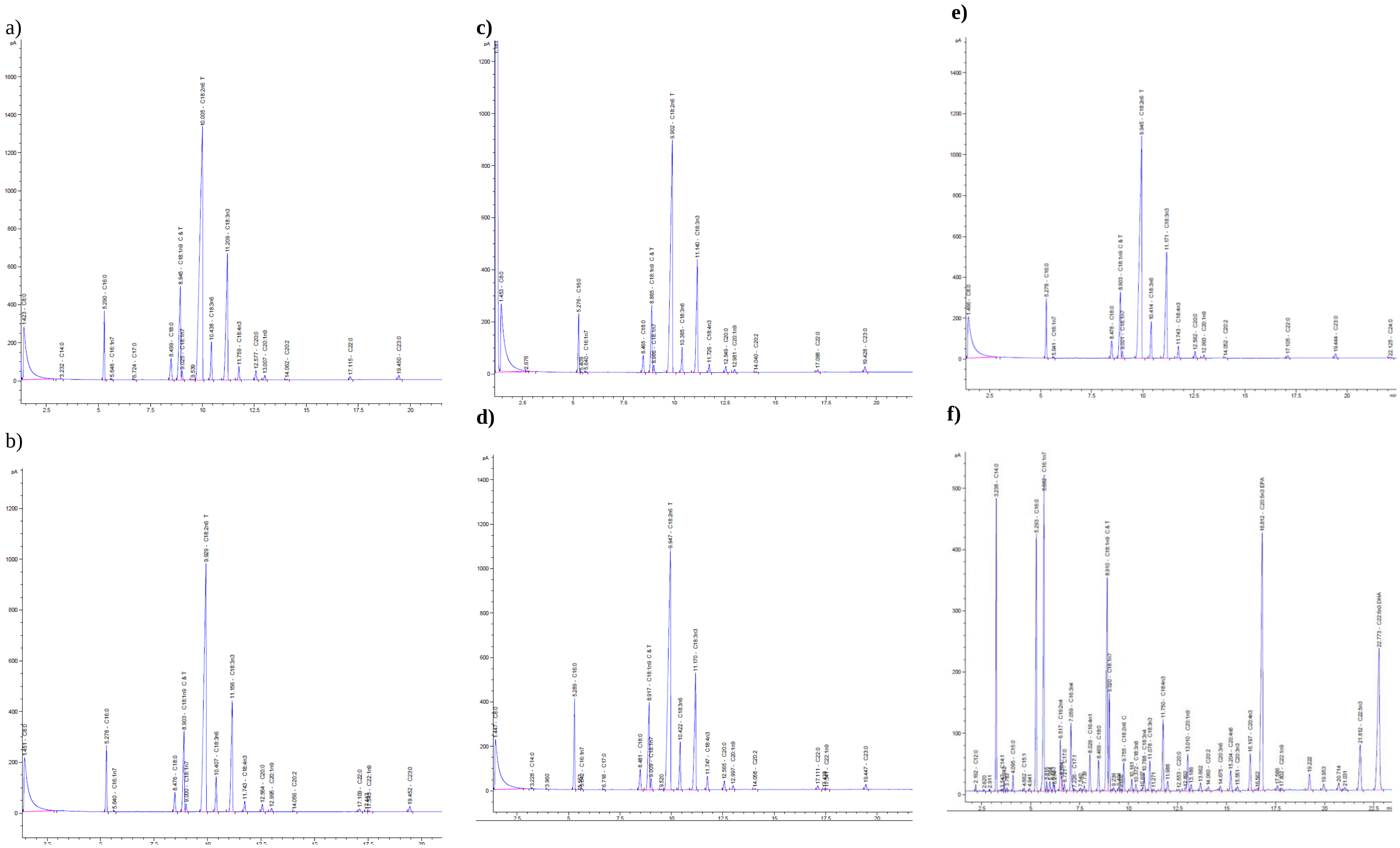

Figure S2. GC-FID of fatty acid analysis of oil extracted from hemp seeds and hemp by-products. a) hemp hearts, b) whole hemp seeds, c) hemp cake, d) hemp hulls, e) cold pressed hemp oil and f) PUFA standards.

a)

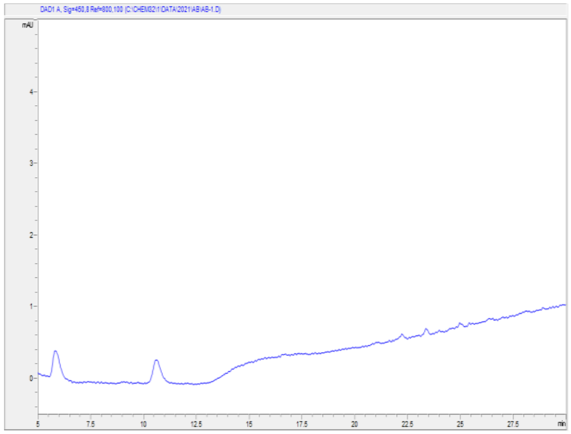

b)

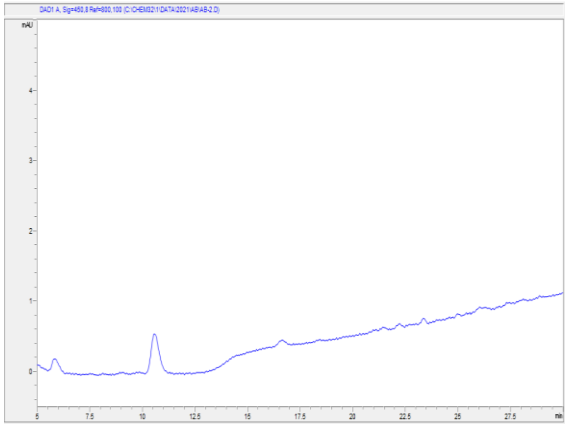

c)

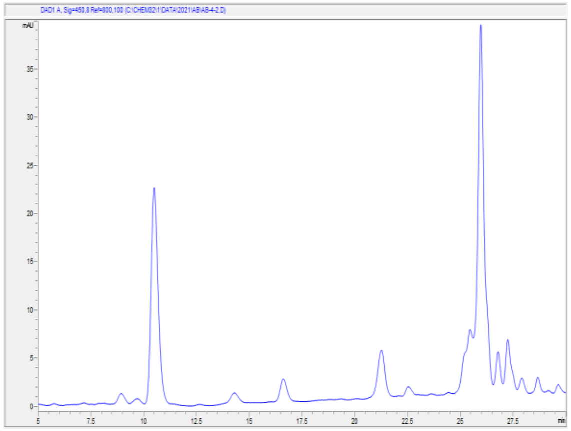

d)

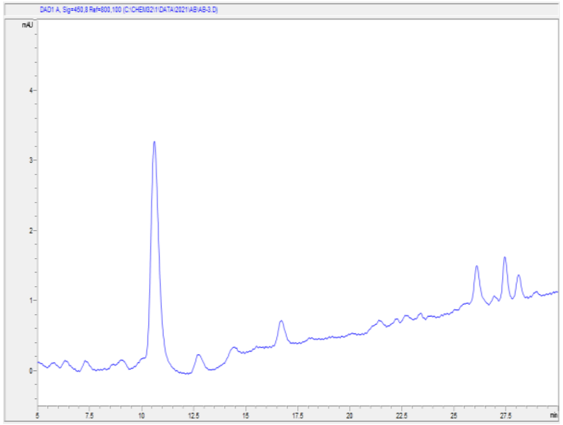

e)

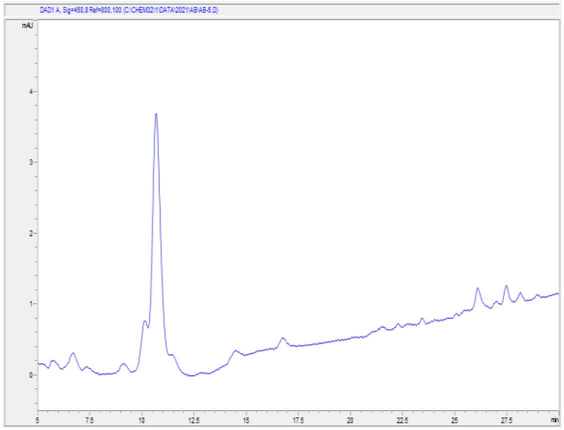

Figure S3. HPLC chromatograms of oils derived from hemp seeds and hemp seed by-products. Retention time lutein – 10.6 min, α-carotene – 26.0 min and β-carotene – 27.4 min. a) hemp hearts, b) whole hemp seeds, c) hemp cake, d) hemp hulls, e) cold pressed hemp oil and f) PUFA standards.

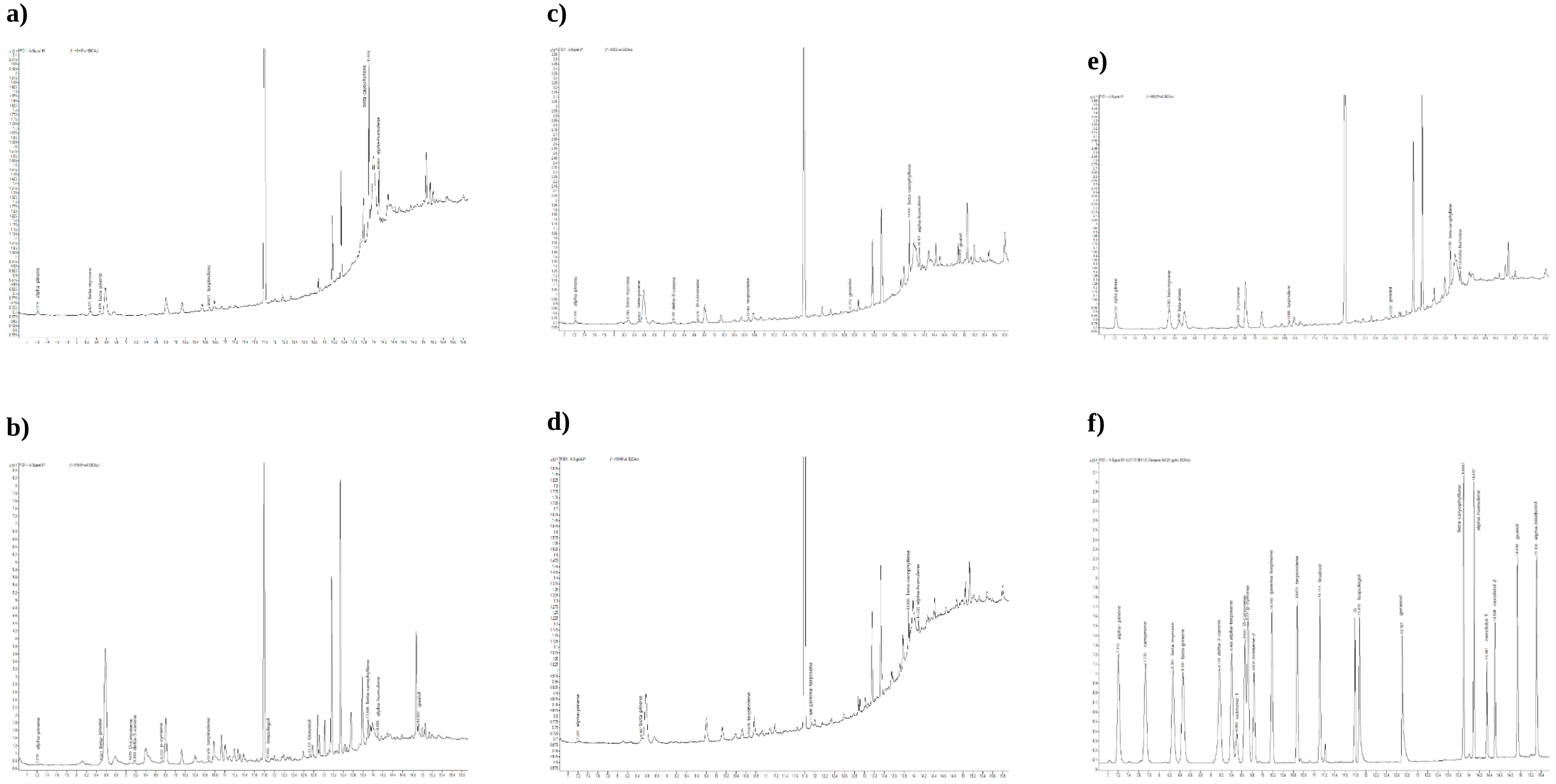

Figure S4. TIC of oils derived from hemp seeds and hemp by-products in GC-MS analysis. a) hemp hearts, b) whole hemp seeds, c) hemp cake, d) hemp hulls e) cold pressed hemp oil and f) standard terpenes.

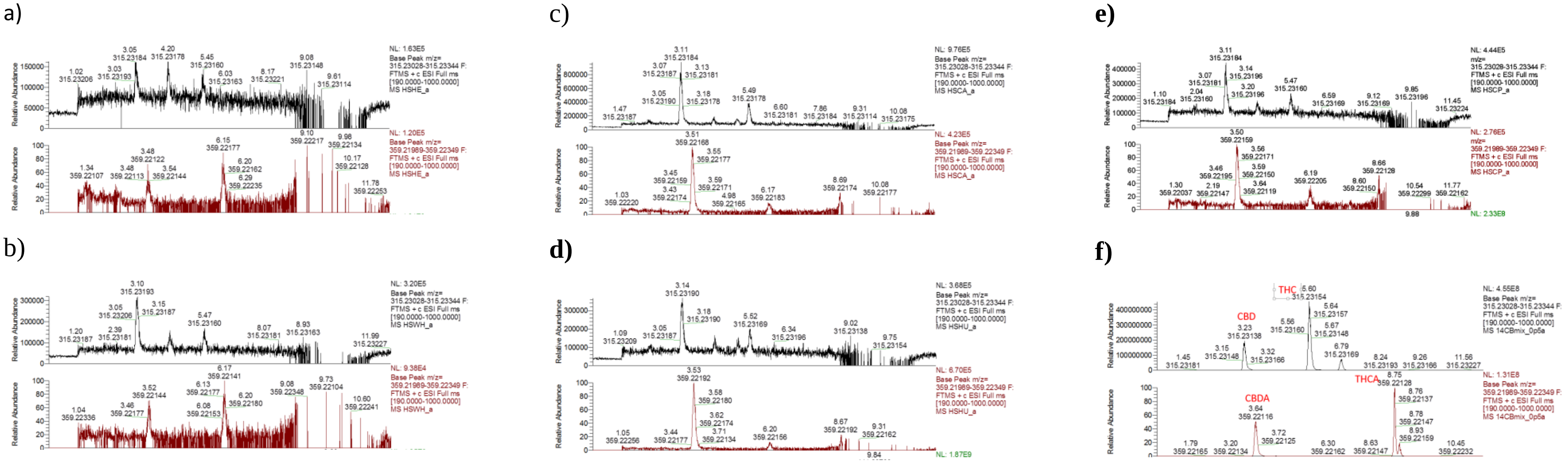

Figure S5. Single ion monitoring of CBD, CBDA, delta-9-trtrahydrocannabinol (THC) and delta-9-trtrahydrocannabinoic acid (THCA). a) hemp hearts, b) whole hemp seeds, c) Hemp cake, d) hemp seed hulls e) cold pressed hemp oil and f) standard terpenes.

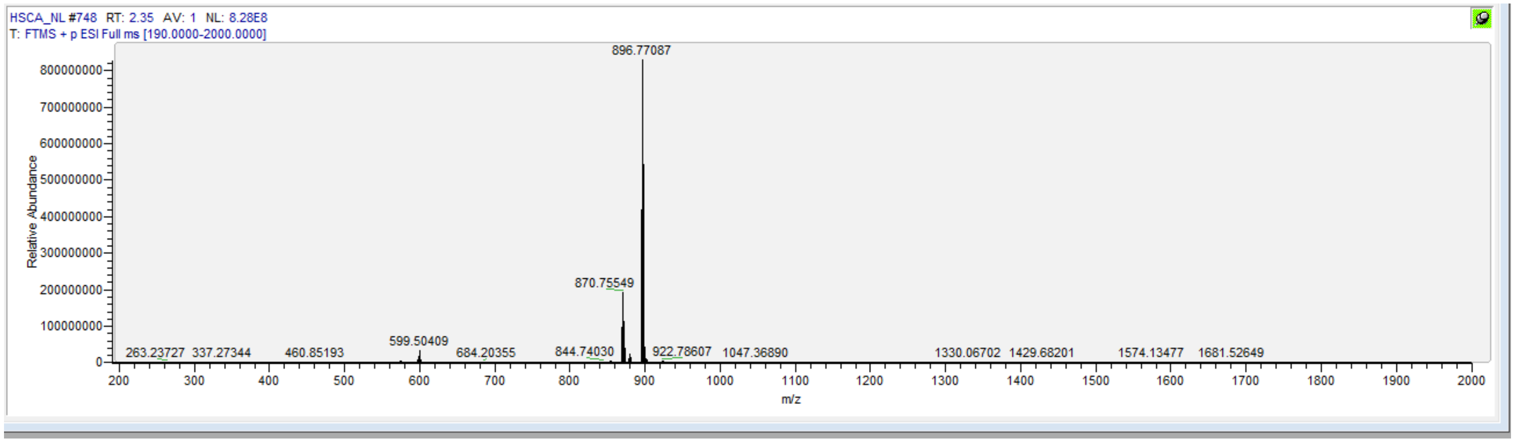

Figure S6. MS spectrum of hemp cake (HSCA) observed at 2.35 min having TAGs ammonium adduct ions. There were minor variations (typically less than 1 ppm mass error) in masses compared to those reported in Table 2, which showed the average across a chromatographic peak.
